# Laparoscopic surgery is associated with reduced long-term reoperation risk in Crohn’s disease

**DOI:** 10.1007/s10151-026-03389-3

**Published:** 2026-07-23

**Authors:** L. Schlager, M. Hamidi, T. Dengler, B. Fallmann, C. Krall, P. Schreiner, W. Reinisch, A. Hansmann, M. Bergmann, A. Stift, L. W. Unger

**Affiliations:** 1 Division of Visceral Surgery, Department of General Surgery, Medical University of Vienna, Währinger Gürtel 18-20, 1090 Vienna, Austria; 2 Department of Medical Statistics, Medical University of Vienna, Vienna, Austria; 3 Division of Gastroenterology and Hepatology, Dept. of Medicine III, Medical University of Vienna, Vienna, Austria; 4 Department of Colorectal Surgery, Oxford University Hospitals NHS Foundation Trust, Oxford, UK

**Keywords:** Ileus, Recurrence, Complications, Inflammatory bowel disease, Laparotomy

## Abstract

**Background:**

Minimally invasive surgery (MIS) improves short-term outcomes in Crohn’s disease (CD), but its impact on long-term surgical recurrence is uncertain.

**Methods:**

The objective of this study was to assess risk factors for surgical recurrence after intestinal resection for CD, with emphasis on surgical approach. A retrospective single-centre study of patients undergoing elective intestinal resection for CD between 1995 and 2020 was conducted. Demographics, disease features, surgical details and complications were analysed. Recurrence was assessed using Kaplan–Meier and Cox regression.

**Results:**

A total of 641 patients were included, with a mean follow-up of 12.4 years. Mean age at surgery was 38.3 years; mean disease duration was 11.5 years. Laparoscopy was performed in 270 patients, and open surgery in 371. Cumulative recurrence was 25% at 25 years. Kaplan–Meier analysis demonstrated lower cumulative surgical recurrence rates following laparoscopic compared with open surgery (log-rank *p* = 0.010). In multivariate Cox regression analysis, open surgery was associated with a higher hazard of surgical recurrence (HR 1.76, 95% CI 1.14–2.69), alongside smoking, female sex and microscopically inflamed resection margins. Severe perioperative complications (7.0% overall; 3.3% laparoscopic versus 9.7% open) did not influence recurrence (*p* = 0.574).

**Conclusions:**

Laparoscopic surgery was associated with longer reoperation-free survival and a lower hazard of surgical recurrence in this long-term cohort. Surgical approach may represent a potentially modifiable factor in optimising long-term outcomes in Crohn’s disease, alongside smoking cessation and achieving microscopically clear resection margins.

**Graphical Abstract:**

**Figure:**
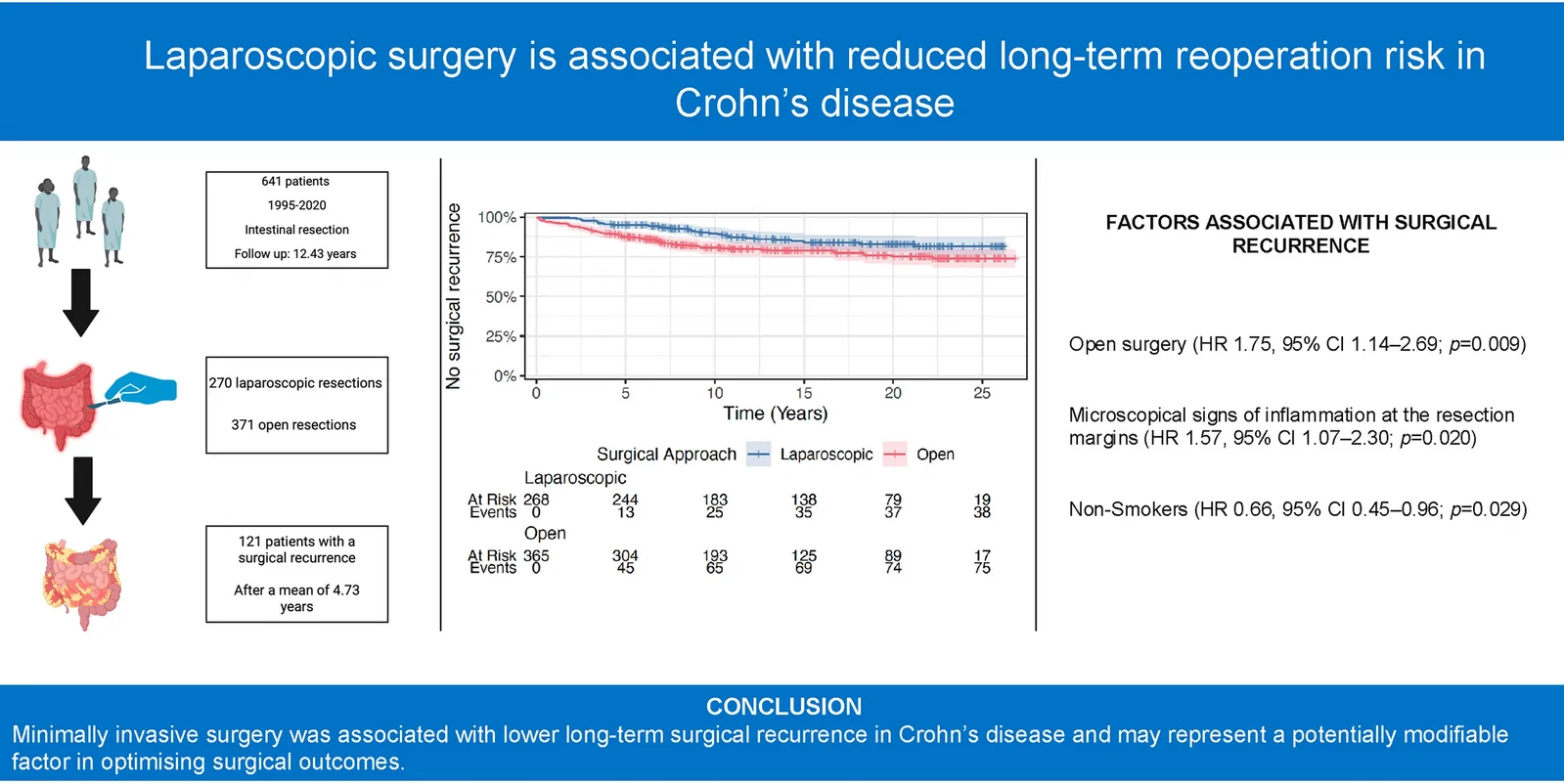

**Supplementary Information:**

The online version contains supplementary material available at https://doi.org/10.1007/s10151-026-03389-3.

## Introduction

Crohn’s disease (CD), an inflammatory bowel disease (IBD) that can affect the entire gastrointestinal tract, continues to present a significant disease burden for patients and healthcare systems [1, 2]. Owing to heterogeneity in disease manifestation, attributed to disease behaviour, disease location and the presence or absence of other complicating factors such as perianal disease or extraintestinal manifestations, treatment is tailored to the specific needs of individual patients. In select cases, a ‘surgery first’ approach may be the right choice for patients with exclusively terminal ileal disease [3, 4], but the availability of several first-line therapies and the reduction of cost for treatments has, in general, resulted in a trend towards early start and broader use of advanced medical therapies [3, 5, 6].

Although early initiation of medical therapy can reduce the requirement for surgery within the first year of diagnosis [6], real-world evidence indicates that the overall rate of decline in surgical intervention is slow, if present at all [7]. As a result, a significant fraction of patients still require such surgical intervention during their lifetime [7, 8]. While the increasing utilisation of minimally invasive surgery is known to improve overall outcomes and reduce inpatient hospital stays and postoperative pain, postoperative complications remain a significant challenge in clinical surgical practice [9–11]. Similar to a large abdominal midline incision for open surgery, complications prolong recovery time, delay the restart or continuation of biologics, necessitate additional invasive interventions and can result in a chronic inflammatory stimulus [8], more severe intraabdominal adhesions, and difficulties in differentiating pain from small incisional hernias or adhesions from obstructive symptoms due to CD recurrence. Although it is acknowledged that laparoscopic surgery significantly reduces both, readmissions as well as reoperations due to ileus in oncological colorectal surgery, such data is currently not available for IBD-related surgery [12].

Therefore, limiting the incisional length and complications to an absolute minimum is a critical surgical objective in managing CD in the long-term, especially as a significant fraction of patients have a complex surgical history [8, 13, 14]. Optimised therapy for patients with CD includes identifying the optimal timing for surgery and warrants multidisciplinary discussions, with novel imaging techniques potentially allowing for a better selection of patients benefiting from surgery rather than medical therapy in e.g. fibrostenotic versus inflammatory disease behaviour [15, 16].

Recurrence in Crohn’s disease can be defined endoscopically, clinically or surgically, and these end points do not necessarily coincide: endoscopic and clinical recurrence are common and frequently amenable to medical therapy, whereas surgical recurrence, the need for further intestinal resection, represents the most clinically meaningful and irreversible end point, and is the focus of this study. Given the limited availability of data comparing long-term benefits and reduced surgical recurrence rates in minimally invasive versus open surgery, we set out to interrogate a dataset spanning 25 years at a tertiary Crohn’s surgery referral centre. We utilised our large single-centre dataset and selected a period allowing for adequate follow-up time as well as a time during which all surgeons performed side-side wide-lumen stapled anastomoses to exclude bias of anastomosis configuration on recurrence rates. Thus, we set out to analyse demographic, disease-related and surgical factors associated with long-term surgical recurrence, with particular focus on surgical approach. Additionally, we include surgical complexity as a variable to identify patients at the highest risk of postoperative complications and recurrence.

## Methods

### Patients

**Fig. 1:**
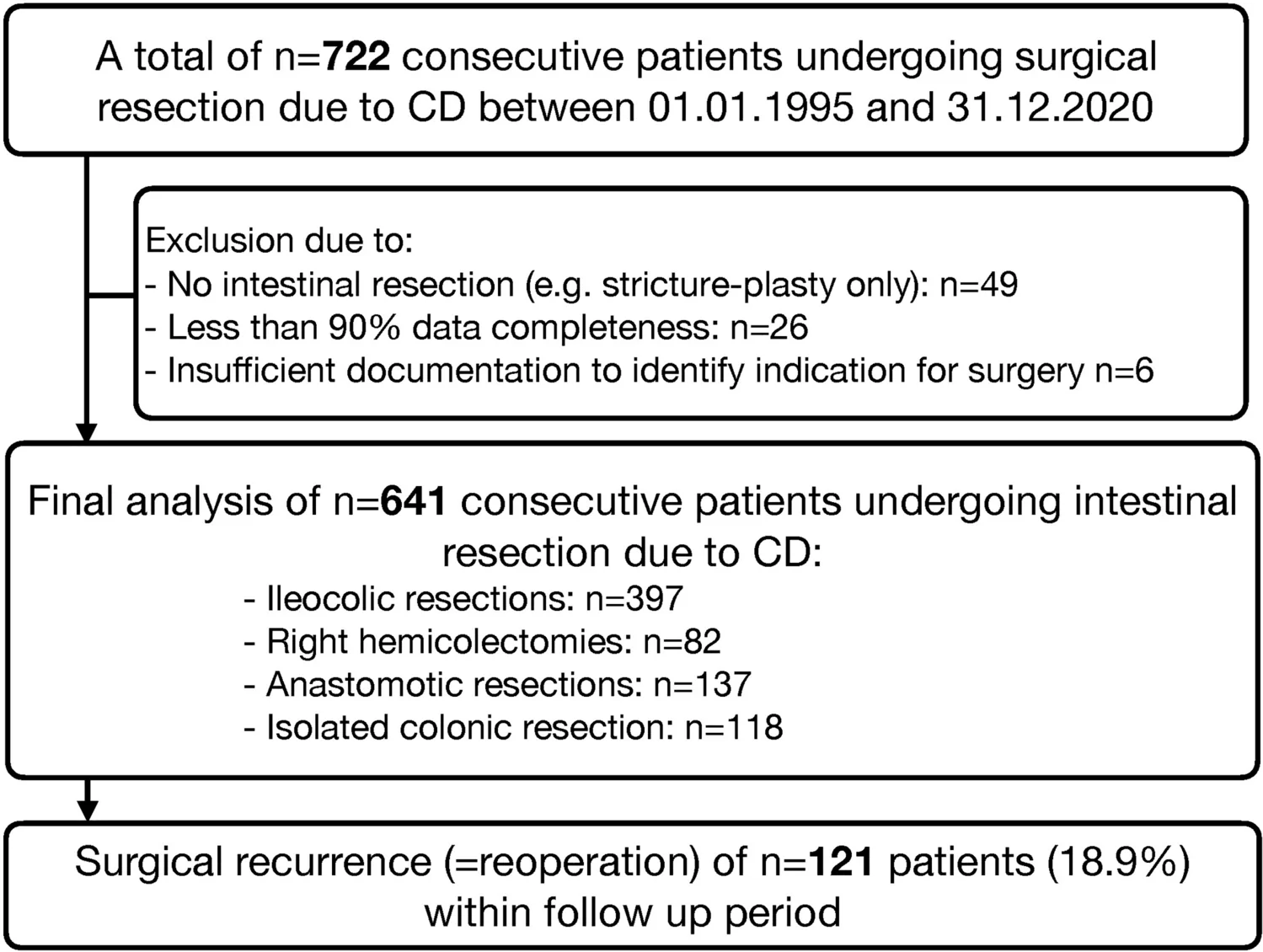
Flowchart of patient inclusion and exclusion

All consecutive patients undergoing surgery due to CD between 1 January 1995 and 31 December 2020, at the Medical University of Vienna’s tertiary care IBD unit in Vienna, Austria, were retrospectively analysed using electronic medical records (Fig. 1). Inclusion criteria were defined as i) established diagnosis of CD, ii) at least one intestinal resection during surgery and iii) 18 years of age at the time of surgery. Exclusion criteria were i) emergency surgery and ii) < 90% data availability of the evaluated parameters (incomplete data).

### Data collection

The study authors extracted data on patient demographics, preoperative comorbidities, perioperative characteristics and postoperative outcomes from electronic medical records. Two independent researchers (M.H., T.D.) collected data, in case of uncertainty in interpreting results (e.g. OP notes), a third researcher (L.S., L.W.U.) was asked to review patient records and a consensus was sought. Two independent researchers (L.S., L.W.U.) conducted quality control by double-checking the raw data of randomly selected patients in 25% of cases. All extracted data were systematically pseudonymised and stored in a secure electronic database for analysis provided by the Medical University of Vienna’s in-house Research, Documentation and Analysis (RDA) system. Access to the database is limited to authorised study personnel only and allows data extraction only in a pseudonymised manner.

The Clavien–Dindo classification (CDC)[17] system, which grades complication severity from 0 (ordinary postoperative course) to 5 (postoperative death), was utilised to evaluate the frequency and severity of complications. No differentiation was made between CDC grade 0 (ordinary postoperative course) and CDC grade 1, as reliable retrospective differentiation between a normal postoperative course or ‘any deviation from a normal postoperative course (CDC grade 1)’ was deemed prone to error.

The choice of surgical approach was based on surgeon expertise, disease extent, prior surgical history and intraoperative findings. Procedures that were commenced laparoscopically but converted to an open approach were classified and analysed within the laparoscopic group on an intention-to-treat basis.

### Objective

The objective of this study was to investigate the impact of demographic factors, disease characteristics and mode of surgery, as well as surgical complexity, on long-term surgical recurrence risk in Crohn’s disease.

Additionally, risk factors for major postoperative complications (CDC > 2) were evaluated.

### Statistical analysis

Data management and statistical analyses were performed using R version 4.5.0.

CDC was dichotomised for analysis: CDC < 2 was classified as no/minor complication, and CDC > 2 as major complication as CDC grade 3 involved invasive procedures (procedure under local anaesthesia or general anaesthesia).

Continuous variables were summarised using mean ± standard deviation (SD), median and first and third quartiles (Q1–Q3). Categorical variables were summarised as counts and percentages.

The number of surgeries over time was plotted, stratified by surgical approach (laparoscopic versus open) and by surgical recurrence status (Supplementary Fig. 1). For the primary outcome of surgical recurrence, Kaplan–Meier curves were generated overall and stratified by surgical approach and other relevant variables. To explore factors associated with major complications (CDC), univariate and multivariate logistic regression models were fitted.

For the time-to-event outcome of surgical recurrence, univariate and multivariate Cox proportional hazards models were used. Regression results are reported as odds ratios (OR) or hazard ratios (HR), as appropriate, with 95% confidence intervals (CI). A *p*-value < 0.05 was considered statistically significant. No adjustment of the *p*-values was performed due to the explorative character of the study.

## Results

During the observational period, 722 patients underwent elective surgery due to CD, and 641 patients were ultimately eligible for analysis (Fig. 1). Eight patients were excluded from survival analyses regarding surgical recurrence due to missing data.

The mean follow-up was 12.4(± 6.8) years. The mean age at the time of surgery was 38 (± 13) years, and the mean disease duration was 11 (± 9) years. Just over half of the patients identified as male (*n *= 343; 54%), and just over 2 in 5 patients (*n* = 274; 43%) were actively smoking at the time of surgery. Patient demographics are shown in Table 1.

**Table 1:** Descriptive overview of patient characteristics, stratified by surgical approach (laparoscopic and open) and surgical recurrence (yes and no)

	All patients	Surgical approach			Surgical recurrence	
	*n* = 641	Laparoscopic *n* = 270	Open *n* = 371	No *n* = 520		Yes *n* = 121
*Disease duration (years)*						
Mean (SD)	11 (9)	8 (8)	14 (9)	12 (9)		11 (9)
Median (Q1, Q3)	10 (4, 17)	6 (1, 12)	13 (7, 19)	10 (3, 18)		10 (5, 15)
Unknown	1	0	1	0		1
*Gender, n (%)*						
Female	298 (46%)	138 (51%)	160 (43%)	230 (44%)		68 (56%)
Male	343 (54%)	132 (49%)	211 (57%)	290 (56%)		53 (44%)
*Smoker at surgery, n (%)*						
Yes	274 (43%)	127 (47%)	147 (40%)	207 (40%)		67 (55%)
No	367 (57%)	143 (53%)	224 (60%)	313 (60%)		54 (45%)
*Age (years)*						
Mean (SD)	38 (13)	35 (13)	41 (13)	39 (13)		37 (12)
Median (Q1, Q3)	37 (28, 47)	32 (24, 43)	39 (31, 50)	37 (28, 48)		36 (28, 43)
*CDC, n (%)*						
Minor complication	596 (93%)	261 (97%)	335 (90%)	484 (93%)		112 (93%)
Major complication	45 (7.0%)	9 (3%)	36 (10%)	36 (7%)		9 (7%)
*BMI*						
Mean (SD)	23.1 (4.8)	22.5 (4.7)	23.5 (4.09)	23.4 (5.3)		21.7 (4.6)
Median (Q1, Q3)	21.2 (18.9, 24.2)	21.8 (19.3, 24.5)	20.8 (18.6, 24.2)	21.3 (19.0, 24.3)		20.7 (18.6, 23.7)
*Postoperative TNFi, n (%)*						
Yes	67 (10.5%)	20 (7.4%)	47 (12.7%)	60 (11.5%)		7 (5.8%)
No	574 (89.5%)	250 (92.6%)	324 (87.3%)	460 (88.5%)		114 (94.2%)
Mean (SD)	167 (73)	149 (53)	180 (82)	166 (72)		170 (75)
Median (Q1, Q3)	155 (115, 200)	137 (110, 180)	170 (120, 225)	150 (115, 195)		165 (110, 215)
*Previous surgeries, n (%)*						
Mean (SD)	0.76 (1.17)	0.26 (0.75)	1.12 (1.28)	0.76 (1.21)		0.72 (1.00)
Median (Q1, Q3)	0.00 (0.00, 1.00)	0.00 (0.00, 0.00)	1.00 (0.00, 2.00)	0.00 (0.00, 1.00)		0.00 (0.00, 1.00)
*Surgical recurrence, n (%)*						
Yes	121 (19%)	40 (14.8%)	81 (21.8%)			
No	520 (81%)	230 (85.2%)	290 (78.2%)			
*Surgical approach, n (%)*						
Laparoscopic	270 (42%)			230 (44%)		40 (33%)
Open	371 (58%)			290 (56%)		81 (67%)
*Anastomoses, n (%)*						
Mean (SD)	0.99 (0.42)	1.00 (0.43)	0.98 (0.52)	0.99 (0.38)		0.99 (0.56)
Median (Q1, Q3)	1.00 (1.00, 1.00)	1.00 (1.00, 1.00)	1.00 (1.00, 1.00)	1.00 (1.00, 1.00)		1.00 (1.00, 1.00)
*Stoma, n (%)*						
1	565 (88%)	263 (97%)	302 (81%)	468 (90%)		97 (80%)
2	74 (12%)	7 (3%)	67 (18%)	50 (9.6%)		24 (20%)
3	2 (0.3%)	0 (0%)	2 (1%)	2 (0.4%)		0 (0%)
*Microscopically positive* *resection margins, n (%)*						
Yes	270 (42%)	95 (35%)	175 (47%)	206 (40%)		64 (53%)
No	371 (58%)	175 (65%)	196 (53%)	314 (60%)		57 (47%)
*Ileocolic resection, n (%)*						
Yes	397 (62%)	213 (79%)	184 (50%)	317 (61%)		80 (66%)
No	244 (38%)	57 (21%)	187 (50%)	203 (39%)		41 (34%)
*Right hemicolectomy, n (%)*						
Yes	82 (13%)	25 (9%)	57 (15%)	63 (12%)		19 (16%)
No	559 (87%)	245 (91%)	314 (85%)	457 (88%)		102 (84%)
*Isolated colonic resection, n (%)*						
Yes	118 (18%)	23 (9%)	95 (26%)	99 (19%)		19 (16%)
No	523 (82%)	247 (91%)	276 (74%)	421 (81%)		102 (84%)
*Anastomotic resection, n (%)*						
Yes	137 (21%)	28 (10%)	109 (29%)	109 (21%)		28 (23%)
No	504 (79%)	242 (90%)	262 (71%)	411 (79%)		93 (77%)
*Strictureplasty, n (%)*						
Mean (SD)	0.25 (0.80)	0.22 (0.83)	0.28 (0.78)	0.24 (0.79)		0.30 (0.85)
Median (Q1, Q3)	0.00 (0.00, 0.00)	0.00 (0.00, 0.00)	0.00 (0.00, 0.00)	0.00 (0.00, 0.00)		0.00 (0.00, 0.00)

*BMI* body mass index, *CDC* Clavien–Dindo classification, TNFi tumour necrosis factor alpha inhibitor

*p*-values depicted in bold show a *p*-value <0.05

Disease behaviour as defined by the Montreal Classification classified the majority of patients as A2 (*n* = 469; 73%), B2 (*n* = 528; 82%) and L3 (*n* = 303; 47%)[18]. At the time of surgery, active perianal disease was documented in only 74 patients (12%). (Supplementary Table 1).

The most commonly performed operation type was ileocolic resection (*n* = 397; 62%), followed by re-do anastomotic resections (*n* = 137; 21%), isolated colonic resections (*n* = 118; 18%), and right hemicolectomies (*n* = 82; 13%). More than half of surgeries were performed using an open surgical approach (*n* = 371; 58%).

The mean duration of surgery was 167 (± 73) min, the mean number of previous surgeries was 0.76 (± 1.17) and the median length of postoperative stay was 8.0 days (IQR 3.0).

### Open surgery and increased surgical complexity are associated with higher complication rates

Overall, the majority of patients (*n* = 596; 93.0%) had no or only minor complications not requiring any invasive treatment (CDC grade 0/I or II; Supplementary Table 1). The most common complication was the requirement for non-standard medicinal products such as antibiotics or blood transfusions (*n* = 89; 13.9%). Overall, 45 (7.0%) patients had major complications requiring invasive interventions (CDC grade III or higher). While only a small proportion of patients underwent interventions requiring local anaesthesia (*n* = 8; 1.2%), 29 patients (4.5%) required a return to theatre. Almost all patients were managed by ward-based care even in the event of complications, and only 6 (0.9%) patients had a single organ dysfunction that required treatment in an intensive care unit. The overall perioperative mortality rate was 0.3%, as two patients died during the postoperative course, one due to a pulmonary embolism, and one due to pulmonary oedema in pre-existing pulmonary hypertension. In line with published literature, patients with major postoperative complications had, on average, more previous surgeries (1.36 ± 2.05 versus 0.71 ± 1.06; *p* < 0.001) and a longer duration of surgery (234 ± 115 versus 162 ± 66 min; *p* < 0.001).

Most importantly, patients who underwent open surgery (10%; 36/371) had a higher rate of major complications than those who underwent laparoscopic surgery (3%; 9/270) (Supplementary Table 2). Examining the specific surgeries performed, patients undergoing ileocecal resection (ICR) had lower percentages of major postoperative complications compared with all other types of resection (5% (18/397) versus 11% (27/244); *p* = 0.002). On the contrary, patients who underwent a re-do procedure of anastomotic resection showed higher percentages of major postoperative complications compared with every other type of resection (12% (16/137) versus 6% (29/504); *p* = 0.016). Thus, to investigate which factors might independently predict complications, we performed uni- and multivariate binary regression analyses (Table 2). Similar to chi^2^ testing, duration of surgery, number of previous surgeries, the number of anastomoses, stoma formation, smoking status at the time of surgery, performed operation and mode of surgery showed an impact on the risk of major complications in univariable testing. Only the duration of surgery remained an independent predictor for postoperative complications with an OR of 1.009 per min (95% CI 1.005–1.013; *p* < 0.001; Table 2).

**Table 2:** Univariable and multivariable binary logistic regression analysis evaluating factors impacting on occurrence of major complications

Logistic regression for major postoperative complications						
	Univariate binary regression			Multivariate binary regression		
Variable	OR	95% CI	*p*	OR	95% CI	*p*
Duration of surgery (minutes)	1.010	1.007–1.014	** < 0.001**	1.009	1.005–1.013	** < 0.001**
Number of anastomoses	0.403	0.198–0.836	**0.013**	0.464	0.190–1.041	0.078
Stoma (number)	2.379	1.140–4.617	**0.014**	0.749	0.266–1.881	0.560
Disease duration (years)	1.059	1.028–1.090	** < 0.001**	0.749	0.982–1.056	0.312
Previous surgeries (number)	1.364	1.125–1.642	**0.001**	0.948	0.728–1.213	0.676
Microscopically positive resection margins	1.791	0.975–3.329	0.061	1.371	0.704–2.678	0.352
Ileocolic resection (none)	2.620	1.420–4.942	**0.002**	1.244	0.603–2.580	0.554
Anastomotic resection	0.462	0.246–0.896	**0.018**	0.512	0.236–1.127	0.091
Surgical approach (Open)	3.116	1.539–6.996	**0.003**	1.549	0.681–3.778	0.312
Smoking status (non-smoker)	1.384	0.745–2.658	0.314	1.176	0.595–2.388	0.645
Age (years)	1.016	0.993–1.038	0.170			
Strictureplasty	1.279	0.942–1.646	0.075			
BMI	0.994	0.934–1.008	0.743			
TNFi (post-operation)	0.608	0.275–1.539	0.250			
Gender (Male)	1.628	0.877–3.126	0.130			
Surgical recurrence (No)	1.080	0.477–2.214	0.842			
Right hemicolectomy (None)	1.543	0.602–5.240	0.420			
Colonic resection	0.595	0.305–1.235	0.142			

*BMI* body mass index, *CI* confidence interval, *OR* odds ratio, TNFi tumour necrosis factor alpha inhibitor

p-values depicted in bold show a *p*-value <0.05

### Open surgery, female sex, smoking and microscopically inflamed resection margins, are associated with earlier surgical recurrence

To investigate the factors associated with long-term recurrence risk, a univariate and multivariate Cox proportional hazards model was used to analyse the time until surgical recurrence. Univariate analysis revealed that the surgical approach, smoking status, gender, and microscopically inflamed resection margins significantly correlated with surgical recurrence risk (Table 3). When performing multivariate analysis, all parameters maintained their significance, thereby proving to be independent predictors of earlier surgical recurrence (Table 3).

**Table 3:** Cox proportional hazards models of time until surgical recurrence

Cox regression for surgical recurrence						
	Univariate regression			Multivariate regression		
Variable	HR	95% CI	*p*	HR	95% CI	*p*
Duration of surgery (min)	1.002	0.999–1.004	0.136	1.002	0.999–1.004	0.222
Surgical approach (Open)	1.665	1.126–2.461	**0.011**	1.756	1.148–2.687	**0.009**
BMI	0.991	0.946–1.019	0.527	0.993	0.971–1.017	0.578
Gender (Male)	0.669	0.462–0.970	**0.034**	0.613	0.419–0.898	**0.012**
Smoking status (non-smoker)	0.663	0.458–0.962	**0.030**	0.659	0.452–0.959	**0.029**
Microscopically positive resection margins	1.718	1.187–2.489	**0.004**	1.574	1.075–2.305	**0.020**
Ileocolic resection (None)	0.951	0.646–1.399	0.798	0.627	0.358–1.098	0.103
Right hemicolectomy (None)	0.662	0.404–1.084	0.101	0.498	0.256–0.972	**0.041**
Anastomotic resection	0.900	0.584–1.388	0.634	0.878	0.503–1.534	0.647
Isolated colonic resection	1.142	0.690–1.890	0.606	1.208	0.640–2.280	0.560
Age at time of surgery (years)	0.995	0.981–1.010	0.517			
Number of anastomoses	0.908	0.558–1.477	0.697			
Strictureplasty	1.097	0.907–1.327	0.339			
Major complication	1.216	0.615–2.402	0.574			
Disease duration (years)	1.006	0.986–1.027	0.554			
Previous surgeries	1.021	0.869–1.200	0.800			
TNFi (post-operation)	1.246	0.576–2.692	0.577			

*BMI* body mass index, *CI* confidence interval, *HR* hazard ratio, *TNFi* tumour necrosis factor alpha inhibitor

p-values depicted in bold show a *p*-value <0.05

Additionally, patients who did not undergo an index right-sided hemicolectomy had lower odds of surgical recurrence compared with other types of resections (HR 0.50, 95% CI 0.26–0.97; *p* = 0.041). Non-smokers at the time of surgery had a lower hazard of recurrence (HR 0.66, 95% CI 0.45–0.96, *p* = 0.029). Perioperative complications did not alter the long-term surgical recurrence risk (Table 3).

Critically, open surgery was independently associated with an earlier surgical recurrence with a HR of 1.76 (95% CI 1.15–2.69; *p* = 0.009) after adjusting for all other variables (Table 3).

**Fig. 2:**
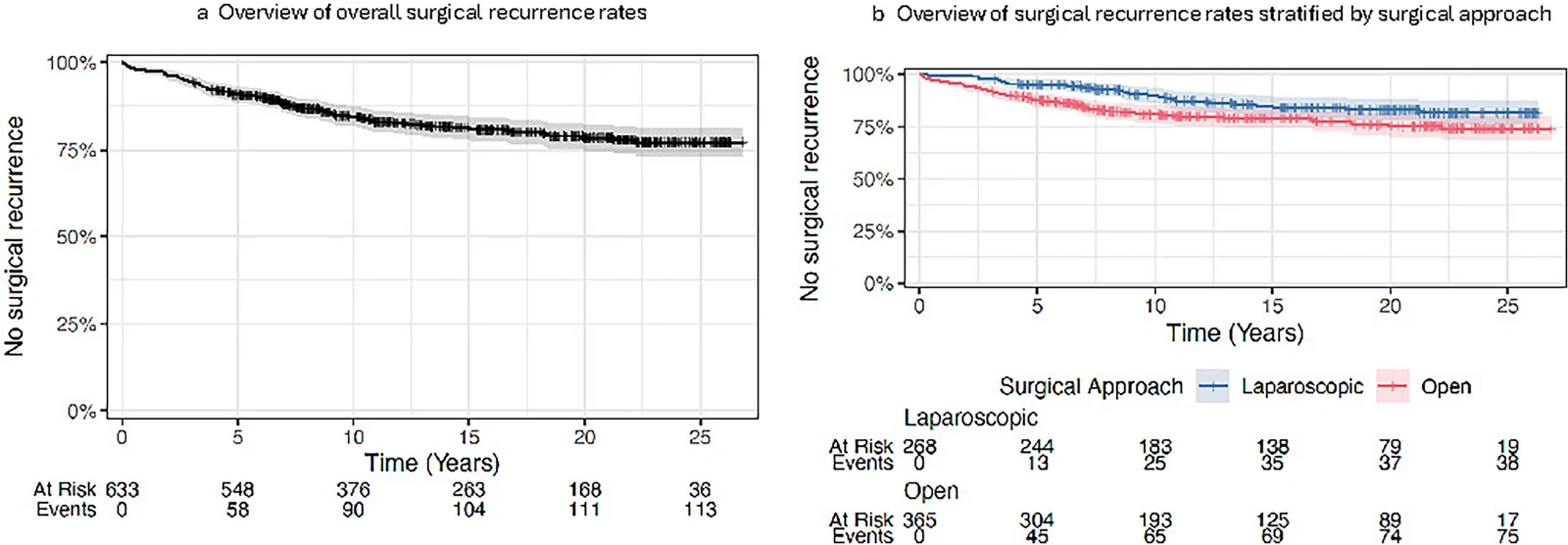
Kalpan–Maier analysis of surgical recurrence rates

**Fig. 3:**
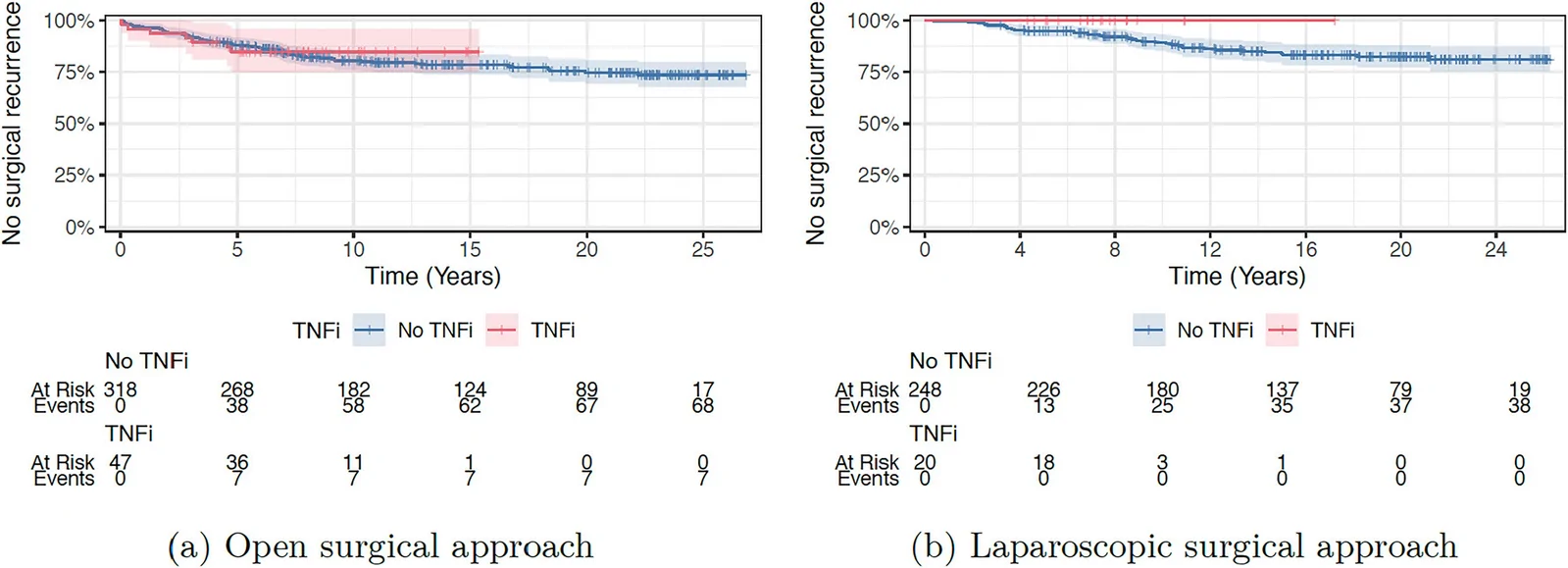
Kalpan–Maier analysis of surgical recurrence stratified by postoperative TNFi therapy

We thus performed Kaplan–Meier analysis of surgical recurrence risk according to operative approach, which showed that the cumulative rate of surgical recurrence increased steadily over time, reaching approximately 15% after 10 years and 25% at 25 years (Fig. 2a). Patients undergoing laparoscopic surgery had significantly lower surgical recurrence rates compared with those treated with open surgery (log-rank, *p* = 0.010; Fig. 2b). Also, male sex, non-smoking and microscopically negative resection margins were associated with lower recurrence rates. Additional Kaplan–Meier analyses were performed to examine the potential effects of TNF inhibition on surgical recurrence. No statistically significant difference in surgical recurrence rates was found in open (log-rank, *p* = 0.59) or laparoscopic (log-rank, *p* = 0.28) surgical approaches when stratified by anti-TNF therapy (Fig. 3).

## Discussion

The findings of this study offer significant insights into the risk factors associated with long-term surgical recurrence in patients undergoing intestinal resections for Crohn’s disease and adds important evidence to current literature, as available studies with shorter follow up have reported no difference in surgical recurrence rates between open and laparoscopic surgery [11, 19]. Patel et al. found a pooled risk ratio for surgical recurrence of 0.78 (95% CI 0.54–1.11; *p* = 0.17) across 870 patients, showing no significant difference between laparoscopic and open surgery [11]. However, interpretation was limited by heterogeneity in follow-up, and the numerical advantage for laparoscopy may reflect sparse long-term data rather than true equivalence. A second meta-analysis by Tan et al. [20], which partially overlapped in included studies, analysed surgical recurrence in only 299 patients, and a relatively brief follow-up period.

In our data, time-to-event analyses using both Kaplan–Meier estimation and Cox proportional hazard modelling consistently demonstrated an association between surgical approach and long-term surgical recurrence. Kaplan–Meier analysis showed a gradual divergence of recurrence-free survival curves over time, with lower cumulative recurrence rates in patients undergoing laparoscopic surgery compared with open surgery. These unadjusted findings were mirrored in multivariate Cox regression, in which open surgery remained associated with a higher hazard of surgical recurrence after adjustment for demographic, disease-related and surgical factors. Importantly, hazard ratios derived from Cox regression describe relative differences in the instantaneous risk of recurrence over time rather than absolute recurrence probabilities and should therefore be interpreted as associations rather than causal effects. While minimally invasive surgery cannot be assumed to directly prevent recurrence, our findings suggest that the surgical approach may represent a potentially modifiable factor associated with longer recurrence-free survival.

Our finding aligns with evidence suggesting that laparoscopic surgery may reduce the postoperative inflammatory response in CD, where patients exhibit a more pronounced response to surgical trauma than those with ulcerative colitis, regardless of surgical access method [21]. Hildebrandt et al. [22] reported lower serum levels of granulocyte elastase after laparoscopic surgery, indicating reduced systemic inflammation, which is relevant given the role of these mediators in driving postoperative complications and recurrence in CD. Our findings add robust long-term data linking minimally invasive surgery to improved outcomes, reinforcing the ECCO-ESCP guidelines, which advocate for MIS whenever feasible [23]. This beneficial effect of limiting inflammation is further underscored by smoking and positive microscopic resection margins, both being known high-risk factors for a sustained inflammatory response, detrimental to mucosal healing [24, 25].

Additionally, all patients undergoing any form of ileocolic anastomosis had their anastomosis done in a standardised fashion with side-to-side, wide-lumen stapled anastomoses, making the anastomotic configuration comparable between groups and eliminating the bias of hand-sewn versus mechanically stapled joins.

Unlike Carvello et al. [26], who linked postoperative complications (particularly Clavien–Dindo > 2) to earlier and increased CD recurrence, we found no association between complication severity and surgical recurrence, with CDC > 2 not predicting recurrence on univariate or multivariate Cox analysis (Table 3). This discrepancy may reflect our cohort of patients undergoing elective surgery, low rate of major complications (7.0%), a different end point and definition of recurrence and prolonged follow-up, during which late recurrences may dilute any early perioperative effect. However, with few major complications, our analysis may be underpowered, and this association warrants further study in larger cohorts.

Postoperative medical prophylaxis also merits consideration. Although stratified Kaplan–Meier analysis showed no significant difference in surgical recurrence by anti-TNF therapy in either open (*p* = 0.59) or laparoscopic (*p* = 0.28) groups, detailed data on biologic type, timing and exposure length is missing. Because laparoscopy was also performed more frequently later, temporal improvements in medical therapy may partly confound the association between surgical approach and recurrence. However, the persistence of the association after multivariable adjustment, alongside the absent anti-TNF effect in our stratified analyses, argues against biologic therapy as the sole explanation but we acknowledge incomplete capture of postoperative biologic exposure as a limitation in our study.

Given the retrospective and explorative design of this study, we do also recognise that patients selected for open surgery, especially in the earlier years of the study period, are likely to have had more complex disease, including a penetrating phenotype, extensive intra-abdominal adhesions and a greater burden of previous abdominal surgery, factors that may simultaneously have favoured an open approach and independently increased the risk of subsequent surgical recurrence. Confounding by indication of this nature cannot be fully excluded in a retrospective design and may contribute to the observed association between open surgery and recurrence. As a single-centre study, findings may not be generalisable to other institutions with varying surgical expertise and perioperative protocols. However, as complication rates remained relatively stable over time (Supplemental Fig. 1) and by only having included patients with > 90% data completeness, we aimed to minimise bias as much as possible. Additionally, the Austrian Electronic Health Record enables viewing of admissions and reoperations for patients even if they were performed in other hospitals, thereby providing a reliable reflection of surgical recurrence.

Future research should focus on optimising perioperative factors, refining laparoscopic techniques to reduce operative time and identifying patients at risk for prolonged procedures to improve overall surgical outcomes. Additionally, we were able to demonstrate that laparoscopic surgery has a reduced risk of surgical recurrence, and emphasise that minimally invasive surgery contributes to reducing inflammation and can further improve long-term outcomes, in addition to smoking cessation and microscopically inflammation-free resection margins.

## Conclusions

In this long-term cohort, laparoscopic surgery was associated with longer recurrence-free survival and a lower hazard of surgical recurrence in CD. Although causal interference is limited by the observational design, the consistency of Kaplan–Meier and Cox regression findings over a 25-year period suggests that the surgical approach may represent a modifiable factor influencing long-term outcomes, alongside established risk factors such as smoking and resection margin inflammation.

## Supplementary Information

Below is the link to the electronic supplementary material.

Supplementary file1 (PDF 195 KB)

Supplementary file2 (PDF 48 KB)

Supplementary file3 (PDF 214 KB)

## Data Availability

All relevant data is shown in tables, figures and supplemental information.
