# Supplementary material for: Laparoscopic surgery is associated with reduced long-term reoperation risk in Crohn’s disease
Source: Tech Coloproctol. 2026 Jul 23;30(1):158. doi: 10.1007/s10151-026-03389-3 (PMC13683536; doi:10.1007/s10151-026-03389-3)
Supplement: Supplementary file 1 [file 10151_2026_3389_MOESM2_ESM.pdf]

MONTREAL CLASSIFICATION (n=641)	
**Montreal A1** (%)	85 (13.2%)
**Montreal A2** (%)	469 (73.2%)
**Montreal A3** (%)	87 (13.6%)
**Montreal L1** (%)	254 (39.6%)
**Montreal L2** (%)	136 (21.2%)
**Montreal L3** (%)	303 (47.3%)
**Montreal L4** (%)	46 (7.2%)
**Montreal B1** (%)	11 (1.7%)
**Montreal B2** (%)	528 (82.4%)
**Montreal B3** (%)	414 (64.6%)
**Montreal p** (%)	74 (11.5%)
**POSTOPERATIVE COMPLICATIONS AS PER CLAVIEN-DINDO CLASSIFICATION (n=641)**	
**0+1** (%)	507 (79.1%)
**2** (%)	89 (13.9%)
**3a** (%)	8 (1.2%)
**3b** (%)	29 (4.5%)
**4a** (%)	6 (0.9%)
**5** (%)	2 (0.3%)

Supplementary Table 1. Montreal Classification and postoperative complications according to the Clavien-Dindo-Classification. Mean±SD
