# Supplementary material for: Laparoscopic surgery is associated with reduced long-term reoperation risk in Crohn’s disease
Source: Tech Coloproctol. 2026 Jul 23;30(1):158. doi: 10.1007/s10151-026-03389-3 (PMC13683536; doi:10.1007/s10151-026-03389-3)
Supplement: Supplementary file 2 [file 10151_2026_3389_MOESM3_ESM.pdf]

| | ALL PATIENTS | CDC | |
|---|---|---|---|
| | n = 641 | CDC ≤2<br>n=596 | CDC > 2<br>n = 45 |
| **Duration of surgery (minutes)** | | | |
| *Mean (SD)* | 167 (73) | 162 (66) | 234 (115) |
| *Median (Q1, Q3)* | 155 (115, 200) | 150 (110, 195) | 215 (150, 285) |
| **Disease duration (years)** | | | |
| *Mean (SD)* | 11 (9) | 11 (9) | 17 (10) |
| *Median (Q1, Q3)* | 10 (4, 17) | 9 (3, 17) | 16 (10, 20) |
| *Unknown* | 1 | 1 | 0 |
| **Gender, n (%)** | | | |
| *Female* | 298 (46%) | 282 (47%) | 16 (36%) |
| *Male* | 343 (54%) | 314 (53%) | 29 (64%) |
| **Smoker at surgery, n (%)** | | | |
| *Yes* | 274 (43%) | 258 (43%) | 16 (36%) |
| *No* | 367 (57%) | 338 (57%) | 29 (64%) |
| **Age** | | | |
| *Mean (SD)* | 38 (13) | 38 (13) | 41 (13) |
| *Median (Q1, Q3)* | 37 (28, 47) | 37 (28, 47) | 38 (29, 47) |
| **CDC, n (%)** | | | |
| *Minor* | 596 (93%) | | |
| *Major* | 45 (7.0%) | | |
| **BMI** | | | |
| *Mean (SD)* | 23.1 (22.9) | 23.2 (23.7) | 21.9 (4.1) |
| *Median (Q1, Q3)* | 21.2 (18.9, 24.2) | 21.2 (18.9, 24.3) | 21.1 (18.6, 23.5) |
| **Duration of surgery (minutes)** | | | |
| *Mean (SD)* | 167 (73) | 162 (66) | 234 (115) |
| *Median (Q1, Q3)* | 155 (115, 200) | 150 (110, 195) | 215 (150, 285) |
| **Previous surgeries, n (%)** | | | |
| *Mean (SD)* | 0.76 (1.17) | 0.71 (1.06) | 1.36 (2.05) |
| *Median (Q1, Q3)* | 0.00 (0.00, 1.00) | 0.00 (0.00, 1.00) | 1.00 (0.00, 2.00) |
| **Surgical recurrence, n (%)** | | | |
| *Yes* | 121 (19%) | 112 (19%) | 9 (20%) |
| *No* | 520 (81%) | 484 (81%) | 36 (80%) |
| **Surgical approach, n (%)** | | | |
| *Laparoscopic* | 270 (42%) | 261 (44%) | 9 (20%) |
| *Open* | 371 (58%) | 335 (56%) | 36 (80%) |
| **Anastomoses, n (%)** | | | |
| *Mean (SD)* | 0.99 (0.42) | 1.00 (0.42) | 0.84 (0.42) |
| *Median (Q1, Q3)* | 1.00 (1.00, 1.00) | 1.00 (1.00, 1.00) | 1.00 (1.00, 1.00) |
| **Stoma, n (%)** | | | |
| *1* | 565 (88%) | 531 (89%) | 34 (76%) |
| *2* | 74 (12%) | 63 (11%) | 11 (24%) |
| *3* | 2 (0.3%) | 2 (0.3%) | 0 (0%) |
| **Microscopically positive resection margins, n (%)** | | | |
| *Yes* | 371 (58%) | 351 (59%) | 20 (44%) |
| *No* | 270 (42%) | 245 (41%) | 25 (56%) |
| **Ileocolic resection, n (%)** | | | |
| *Yes* | 397 (62%) | 379 (64%) | 18 (40%) |
| *No* | 244 (38%) | 217 (36%) | 27 (60%) |
| **Right hemicolectomy, n (%)** | | | |
| *Yes* | 82 (13%) | 78 (13%) | 4 (8.9%) |
| *No* | 559 (87%) | 518 (87%) | 41 (91%) |
| **Isolated colonic resection, n (%)** | | | |
| *Yes* | 118 (18%) | 106 (18%) | 12 (27%) |
| *No* | 523 (82%) | 490 (82%) | 33 (73%) |
| **Anastomotic resection, n (%)** | | | |
| *Yes* | 137 (21%) | 121 (20%) | 16 (36%) |
| *No* | 504 (79%) | 475 (80%) | 29 (64%) |
| **Strictureplasty, n (%)** | | | |
| *Mean (SD)* | 0.25 (0.80) | 0.24 (0.78) | 0.47 (1.01) |
| *Median (Q1, Q3)* | 0.00 (0.00, 0.00) | 0.00 (0.00, 0.00) | 0.00 (0.00, 0.00) |

Supplementary table 2. Descriptive overview of patient characteristics, stratified by complications (classified by CDC)

*Abbreviatons:* BMI body mass index; CDC Clavien Dindo classification
